# Chemotherapy-induced immunogenic modulation of tumor cells enhances killing by cytotoxic T lymphocytes and is distinct from immunogenic cell death

**DOI:** 10.1186/2051-1426-1-S1-P91

**Published:** 2013-11-07

**Authors:** Andressa A Smith, James Hodge, Charlie Garnett, Benedetto Farsaci, Claudia Palena, Kwong-Yok Tsang, Soldano Ferrone, Sofia Gamiero

**Affiliations:** 1 Laboratory of Tumor Immunology and Biology, National Cancer Institute, Bethesda, MD, USA; 2 Cancer Immunology Program, University of Pittsburgh, Pittsburgh, PA, USA

## 

Certain chemotherapeutic regimens trigger cancer cell death while inducing dendritic cell maturation and subsequent immune responses. However, chemotherapy-induced immunogenic cell death (ICD) has thus far been restricted to select agents. In contrast, several chemotherapeutic drugs modulate antitumor immune responses, despite not inducing classic ICD. In addition, in many cases tumor cells do not die after treatment. Here, using docetaxel, one of the most widely used cancer chemotherapeutic agents, as a model, we examined phenotypic and functional consequences of tumor cells that do not die from immunogenic cell death. Docetaxel treatment of tumor cells did not induce ATP or HMGB1 secretion, or cell death. However, calreticulin exposure was observed in all cell lines examined after chemotherapy treatment. Killing by CEA, MUC-1, or PSA-specific CD8+ CTLs was significantly enhanced after docetaxel treatment. This killing was associated with increases in components of antigen-processing machinery, and mediated largely by calreticulin membrane translocation, as determined by functional knockdown of calreticulin, PERK, or calreticulin-blocking peptide. A docetaxel-resistant cell line was selected (MDR-1+, CD133+) by continuous exposure to docetaxel. These cells, while resistant to direct cytostatic effects of docetaxel, were not resistant to the chemomodulatory effects that resulted in enhancement of CTL killing. Here, we provide an operational definition of “immunogenic modulation,” where exposure of tumor cells to nonlethal/sublethal doses of chemotherapy alters tumor phenotype to render the tumor more sensitive to CTL killing. These observations are distinct and complementary to immunogenic cell death and highlight a mechanism whereby chemotherapy can be used in combination with immunotherapy.

